# Nirmatrelvir or Molnupiravir Use and Severe Outcomes From Omicron Infections

**DOI:** 10.1001/jamanetworkopen.2023.35077

**Published:** 2023-09-21

**Authors:** Dan-Yu Lin, Francois Abi Fadel, Shuaiqi Huang, Alex T. Milinovich, Gretchen L. Sacha, Patricia Bartley, Abhijit Duggal, Xiaofeng Wang

**Affiliations:** 1 Department of Biostatistics, Gillings School of Global Public Health, University of North Carolina, Chapel Hill; 2 Department of Pulmonary Medicine, Cleveland Clinic, Cleveland, Ohio; 3 Department of Quantitative Health Sciences, Cleveland Clinic, Cleveland, Ohio; 4 Department of Pharmacy, Cleveland Clinic, Cleveland, Ohio; 5 Department of Infectious Disease, Cleveland Clinic, Cleveland, Ohio; 6 Department of Critical Care Medicine, Cleveland Clinic, Cleveland, Ohio

## Abstract

**Importance:**

Ritonavir-boosted nirmatrelvir and molnupiravir are currently used in the US and in other countries to treat nonhospitalized patients who have mild-to-moderate COVID-19 and who are at high risk for progression to severe disease. The associations of these 2 oral antiviral drugs with hospitalization and death resulting from infection with new SARS-CoV-2 Omicron subvariants, particularly BQ.1.1 and XBB.1.5, are unknown.

**Objective:**

To assess the association of nirmatrelvir or molnupiravir use with the risks of hospitalization and death among patients infected with new Omicron subvariants.

**Design, Setting, and Participants:**

This was a cohort study of patients who received a diagnosis of COVID-19 at Cleveland Clinic from April 1, 2022, to February 20, 2023 (during which the Omicron variant evolved from BA.2 to BA.4/BA.5, then to BQ.1/BQ.1.1, and finally to XBB/XBB.1.5) and who were at high risk of progressing to severe disease, with follow-up through 90 days after diagnosis. The final date for follow-up data collection was February 27, 2023.

**Exposures:**

Treatment with ritonavir-boosted nirmatrelvir or molnupiravir.

**Main Outcomes and Measures:**

The primary outcome was time to death. The secondary outcome was time to either hospitalization or death. The association of either nirmatrelvir or molnupiravir use with each outcome was measured by the hazard ratio (HR) adjusted for demographic factors, socioeconomic status, date of COVID-19 diagnosis, coexisting medical conditions, COVID-19 vaccination status, and previous SARS-CoV-2 infection.

**Results:**

There were 68 867 patients (29 386 [42.7%] aged ≥65 years; 26 755 [38.9%] male patients; 51 452 [74.7%] non-Hispanic White patients). Thirty of 22 594 patients treated with nirmatrelvir, 27 of 5311 patients treated with molnupiravir, and 588 of 40 962 patients who received no treatment died within 90 days of Omicron infection. The adjusted HRs of death were 0.16 (95% CI, 0.11-0.23) for nirmatrelvir and 0.23 (95% CI, 0.16-0.34) for molnupiravir. The adjusted HRs of hospitalization or death were 0.63 (95% CI, 0.59-0.68) for nirmatrelvir and 0.59 (95% CI, 0.53-0.66) for molnupiravir. The associations of both drugs with both outcomes were observed across subgroups defined by age, race and ethnicity, date of COVID-19 diagnosis, vaccination status, previous infection status, and coexisting conditions.

**Conclusions and Relevance:**

These findings suggest that the use of either nirmatrelvir or molnupiravir is associated with reductions in mortality and hospitalization in patients infected with Omicron, regardless of age, race and ethnicity, virus strain, vaccination status, previous infection status, or coexisting conditions. Both drugs can, therefore, be used to treat nonhospitalized patients who are at high risk of progressing to severe COVID-19.

## Key Points

**Question:**

What outcomes are associated with ritonavir-boosted nirmatrelvir or molnupiravir use for outpatient treatment of SARS-CoV-2 Omicron subvariants, particularly BQ.1.1 and XBB.1.5, in high-risk individuals?

**Findings:**

In a cohort study of 68 867 patients who received a diagnosis of COVID-19 at Cleveland Clinic from April 1, 2022, to February 20, 2023, and who were at high risk of progressing to severe COVID-19, both nirmatrelvir and molnupiravir use were significantly associated with reductions in hospitalization and death. The association was observed across subgroups defined by age, race and ethnicity, date of diagnosis, vaccination status, previous infection status, and coexisting conditions.

**Meaning:**

These findings suggest that both nirmatrelvir and molnupiravir can be used to treat nonhospitalized patients who are at high risk of progressing to severe COVID-19.

## Teaser

This cohort study assess the association of nirmatrelvir or molnupiravir use with the risks of hospitalization and death among patients infected with new SARS-CoV-2 Omicron subvariants.

## Introduction

On December 22 and 23, 2021, the US Food and Drug Administration issued Emergency Use Authorization for ritonavir-boosted nirmatrelvir (Paxlovid; hereafter referred to as *nirmatrelvir*) and molnupiravir (Lagevrio), respectively, to treat mild-to-moderate COVID-19 in patients who are at high risk of progressing to severe disease.^1,2^ The clinical trials^3,4^ that established the efficacy of these 2 oral antiviral drugs were conducted in patients who were unvaccinated when the B.1.617.2 (Delta) variant was predominant. An observational study^5^ from Israel showed that the rates of hospitalization and death from the BA.1 (Omicron) variant over 35 days were significantly lower among patients aged 65 years or older who received nirmatrelvir than among those who did not, but no evidence of benefit was found in younger adults. An observational study^6^ from Hong Kong showed that nirmatrelvir and molnupiravir were both associated with reduced risks of hospitalization and death from the Omicron subvariant BA.2.2 in a largely unvaccinated and previously uninfected population of patients older than 60 years. A randomized clinical trial^7^ from the United Kingdom did not find molnupiravir to be effective against hospitalization or death from the Omicron subvariants BA.1 and BA.2 among vaccinated adults with relatively low risk of progressing to severe illness. To our knowledge, the effectiveness of nirmatrelvir or molnupiravir against newer Omicron subvariants, particularly BQ.1.1 and XBB.1.5, has not been investigated.

We conducted a large cohort study using the electronic medical records of Cleveland Clinic patients who received a diagnosis of COVID-19 between April 1, 2022, and February 20, 2023, when the Omicron variant evolved from BA.2 to BA.4/ BA.5, then to BQ.1/BQ.1.1, and finally to XBB/XBB.1.5. We assessed the association of either nirmatrelvir or molnupiravir use with the risks of hospitalization and death from new Omicron subvariants over 90 days among patients who met the treatment eligibility criteria, and we performed subgroup analyses by age, race and ethnicity, date of diagnosis, vaccination status, previous infection status, and coexisting conditions.

## Methods

### Study Design

This observational cohort study used electronic health records in the Cleveland Clinic Health System. Cleveland Clinic is a nonprofit, US academic medical center based in Cleveland, Ohio. It runs a large hospital in Cleveland, 11 affiliated regional hospitals and 19 family health centers in northeast Ohio, and 5 hospitals in Florida. The Cleveland Clinic patients who received a diagnosis of COVID-19 between April 1, 2022, and February 20, 2023, were assessed for eligibility to receive treatments in the outpatient setting. Eligible patients were monitored for up to 90 days to ascertain their clinical outcomes, with February 27, 2023, as the final day of follow-up. During the study period, the primary circulating strains in the study areas changed from BA.2 to BA.4/BA.5, then to BQ.1/BQ.1.1, and finally to XBB/XBB.1.5.

The study was approved by the Cleveland Clinic institutional review board. The requirement for written informed consent was waived because the data were anonymous, in accordance with 45 CFR §46. We followed the Strengthening the Reporting of Observational Studies in Epidemiology (STROBE) reporting guideline for cohort studies.

### Treatments

Nirmatrelvir and molnupiravir were available at Cleveland Clinic beginning on January 11 and 13, 2022, respectively. eFigure 1 in Supplement 1 shows the outpatient COVID-19 therapy decision tree. Eligibility for receiving antiviral drugs took into account drug interactions and other contraindications. Patients with contraindications to nirmatrelvir were offered treatment with molnupiravir or bebtelovimab. We focused on nirmatrelvir and molnupiravir in this study because bebtelovimab is no longer authorized by the Food and Drug Administration.^8^

Nirmatrelvir and molnupiravir were prescribed over the telephone or during virtual visits to local Cleveland Clinic outpatient pharmacies. Cleveland Clinic provided free home delivery through the Cleveland Clinic mail-order pharmacy to make drugs accessible to all patients. Virtually all prescriptions were filled. Both drugs were initiated as soon as possible following COVID-19 diagnosis and within 5 days of symptom onset. For patients with normal kidney function, the dosage was 300 mg of nirmatrelvir with 100 mg of ritonavir taken together orally twice daily for 5 days. For patients with moderately reduced kidney function (estimated glomerular filtration rate [eGFR], 30-59 mL/min), the dosage of nirmatrelvir was reduced to 150 mg. For patients with eGFR less than 30 mL per minute or with severe hepatic impairment (Child-Pugh class C disease), nirmatrelvir was not prescribed. The dosage of molnupiravir was 800 mg taken every 12 hours for 5 days.

### Clinical Outcomes

The primary outcome measure was time to death after COVID-19 diagnosis, and the secondary outcome measure was time to hospitalization or death, whichever occurs first. Patients were monitored for up to 90 days after the diagnosis of COVID-19 or until the end of the study (February 27, 2023), whichever came first. The information on hospitalization and death was obtained from the electronic health records. The records on death were synchronized with the Social Security death master file maintained by the US Social Security Administration and with the vital records from the Ohio Department of Health.

### Inclusion and Exclusion Criteria

Patients with COVID-19 aged 12 years or older were assessed as being at high risk for progression to severe disease. COVID-19 was diagnosed by polymerase chain reaction or antigen test, with the vast majority (>90%) of positive tests being confirmed by polymerase chain reaction at Cleveland Clinic laboratories. The definition of high-risk patients was based on the Cleveland Clinic COVID-19 outpatient treatment guidelines, which followed the National Institutes of Health (NIH) definition (eTable 1 in Supplement 1).

We excluded patients who weighed less than 40 kg, who received bebtelovimab or remdesivir, or who tested positive for COVID-19 after hospitalization. Nirmatrelvir and molnupiravir were not widely available until April 2022 (eFigure 2 in Supplement 1); therefore, we excluded patients who received a diagnosis of COVID-19 before April 1. For the study of each treatment, we excluded the patients who received the other treatment. The control group for the nirmatrelvir study also excluded patients with eGFR less than 30 mL per minute or Child-Pugh class C disease. The control group for the molnupiravir study also excluded patients younger than 18 years and pregnant women.

### Data Collection

Cleveland Clinic established a COVID-19 registry database in March 2020 to align data collection for research with the clinical care of all COVID-19 patients.^9^ Data capture was facilitated by creating standardized clinical templates with an in-house data repository implemented across the health care system. Data were collected and managed using Research Electronic Data Capture tools. Registry variables were chosen to reflect the available literature on COVID-19 disease characterization, treatments, and clinical outcomes.

Patients’ demographic variables, comorbidities, presenting symptoms, and medications were retrieved. Race and ethnicity were self-reported with fixed categories (Hispanic, non-Hispanic Black, non-Hispanic White, other [ie, American Indian, Alaska Native, Asian, Native Hawaiian, Pacific Islander, and multiracial], and unknown) and were collected as part of the US Department of Health and Human Services COVID-19 laboratory reporting requirements. Several common comorbidities were extracted from the electronic health record system (eTable 2 in Supplement 1). Because complete medical histories had been obtained for all patients in the database regardless of the nature of their relationship with Cleveland Clinic, the capture of comorbidities was uniform between treated and untreated patients. Data on vaccination were synchronized with records from pharmacies providing vaccines (ie, CVS, Walgreen, and RiteAid). Patients were considered boosted if they had received a third dose of a COVID-19 vaccine more than 7 days before the COVID-19 diagnosis. Positive tests for SARS-CoV-2 more than 90 days before the current COVID-19 diagnosis were treated as previous infections. Finally, we created a socioeconomic index according to median income, fraction of vacant housing, fraction of poverty, fraction of no health insurance, fraction of high school degree or higher, and fraction of assisted income by the zip code, with a higher index indicating worse socioeconomic well-being.^10^

### Statistical Analysis

We investigated the use of nirmatrelvir and molnupiravir separately among the patients who were eligible for each treatment. We also performed subgroup analyses according to the patient’s age (≥65 vs <65 years), race and ethnicity (non-Hispanic White vs all other categories), date of COVID-19 diagnosis (April 1 to June 21, 2022, BA.2 predominance; June 22 to November 30, 2022, BA.4/BA.5 predominance; and December 1, 2022, to February 20, 2023, BQ.1.1/XBB.1.5 predominance), vaccination status, previous infection status, and coexisting conditions.

For each treatment and each subgroup, we compared the cumulative incidence of death between treated and untreated patients by the Kaplan-Meier estimator. In addition, we estimated the association of treatment with the risk of death by fitting the Cox regression model with a time-dependent treatment variable. For each patient, time 0 was the date when the patient received a diagnosis of COVID-19. For a treated patient, the time-dependent treatment variable took the value 0 before the patient received the treatment and took the value 1 afterward. For an untreated patient, the treatment variable took the value 0 throughout the study. We included the baseline demographic and clinical characteristics as time-independent covariates. Finally, we fit the same model to the composite end point of hospitalization and death. All analyses were performed with R statistical software version 4.2.0 (R Project for Statistical Computing).

## Results

### Patient Population

**Figure 1.**
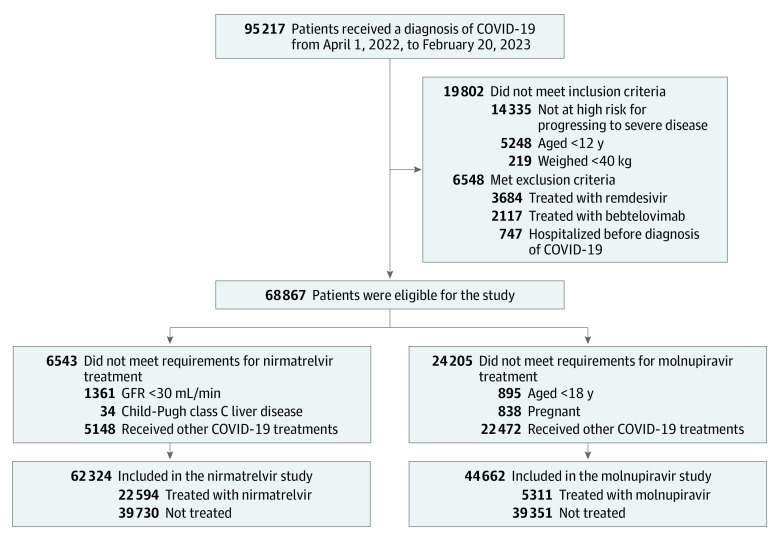
Patient Enrollment Flowchart Patients who received a diagnosis of COVID-19 from April 1, 2022, to February 20, 2023 were assessed for eligibility to receive treatments in the outpatient setting. Patients who received nirmatrelvir or molnupiravir were selected. Patients who were eligible for each treatment but did not receive any treatment were selected as the comparison group for the corresponding treatment group. GFR indicates glomerular filtration rate.

Figure 1 shows the selection of patients for the study. A total of 95 217 patients received a diagnosis of COVID-19 at Cleveland Clinic between April 1, 2022, and February 20, 2023. Among the 68 867 patients who met the study eligibility criteria, 29 386 (42.7%) were aged 65 years or older, 26 755 (38.9%) were male, and 51 452 (74.7%) were non-Hispanic White; 22 594 received nirmatrelvir, and 5311 received molnupiravir. The uptake of these 2 drugs increased steadily over time as the supply increased (eFigure 2 in Supplement 1).

Table 1 shows the characteristics of the patient population. All variables were complete except that race and ethnicity had 1177 missing values, which were included in the other or unknown category. Most COVID-19 cases were diagnosed between June 22, 2022, and February 20, 2023, during which BA.4/BA.5, BQ.1/BQ.1.1, and XBB/XBB.1.5 were circulating. The patients who received nirmatrelvir (mean [SD] age, 61.8 [15.3] years; 18 571 [82%] non-Hispanic White individuals), had a booster vaccination rate of 75% (16 966 patients) and mean (SD) socioeconomic index of 0.30 (0.09), whereas the patients who were eligible for nirmatrelvir but received no treatment (mean [SD] age, 54.0 [19.8] years; 27 542 [69%] non-Hispanic White individuals) had a booster vaccination rate of 50% (19 795 patients) and mean (SD) socioeconomic index of 0.33 (0.11). Similar differences were observed between the patients who received molnupiravir and those who were eligible for molnupiravir but received no treatment. There were also some differences between treated and untreated patients in terms of sex, prior infection, and coexisting conditions (eTable 3 in Supplement 1). We accounted for the unbalances of baseline characteristics by including them as covariates in the regression model. As of February 27, 2023, a total of 645 patients had died (30 of 22 594 patients treated with nirmatrelvir, 27 of 5311 patients treated with molnupiravir, and 588 of 40 962 patients who received no treatment), and 5368 had been hospitalized.

**Table 1.** Demographic and Clinical Characteristics of the Patient Population

Characteristic	Patients, No. (%)					
	Nirmatrelvir		Molnupiravir		Deaths (n = 645)	Hospitalization (n = 5368)
	Treated (n = 22 594)	Untreated (n = 39 730)^a^	Treated (n = 5311)	Untreated (n = 39 351)^a^		
Age, mean (SD), y	61.8 (15.3)	54.0 (19.8)	65.8 (14.6)	55.8 (19.1)	76.8 (13.7)	62.0 (18.3)
Age group, y						
12-49	4716 (21)	16 483 (42)	757 (14)	14 957 (38)	25 (4)	1302 (24)
50-64	6575 (29)	9313 (23)	1328 (25)	9537 (24)	84 (13)	1280 (24)
65-74	6781 (30)	7209 (18)	1704 (32)	7547 (19)	141 (22)	1323 (25)
≥75	4522 (20)	6725 (17)	1522 (29)	7310 (19)	395 (61)	1463 (27)
Sex						
Female	13 581 (60)	25 107 (63)	2799 (53)	24 457 (62)	290 (45)	3360 (63)
Male	9013 (40)	14 623 (37)	2512 (47)	14 894 (38)	355 (55)	2008 (37)
Race and ethnicity						
Hispanic	967 (4)	2623 (7)	178 (3)	2512 (6)	16 (3)	212 (4)
Non-Hispanic Black	1556 (7)	6292 (16)	320 (6)	6357 (16)	90 (14)	739 (14)
Non-Hispanic White	18 571 (82)	27 542 (69)	4499 (85)	27 379 (70)	493 (76)	4094 (76)
Other or unknown^b^	1500 (7)	3273 (8)	314 (6)	3103 (8)	46 (7)	323 (6)
Ohio residency	19 609 (87)	34 325 (86)	4637 (87)	33 789 (86)	566 (88)	4931 (92)
SARS-CoV-2 immunity status						
Vaccine boosters	16 966 (75)	19 795 (50)	4162 (78)	20 012 (51)	326 (51)	3168 (59)
Previously infected	1816 (8)	5131 (13)	444 (8)	5023 (13)	52 (8)	628 (12)
Coexisting conditions						
Respiratory	3994 (18)	7629 (19)	1242 (23)	7696 (20)	208 (32)	1693 (32)
Immunocompromised	1339 (6)	2288 (6)	960 (18)	2889 (7)	282 (44)	1038 (19)
Cardiovascular system	9472 (42)	14 715 (37)	3045 (57)	15 663 (40)	528 (82)	3441 (64)
Diabetes	3263 (14)	5316 (13)	1215 (23)	5865 (15)	235 (36)	1362 (25)
Obesity	3255 (14)	5656 (14)	901 (17)	5721 (15)	131 (20)	1360 (25)
Other nonrespiratory	10 200 (45)	18 040 (45)	3143 (59)	18 570 (47)	563 (87)	4073 (76)
Socioeconomic index score, mean (SD)	0.30 (0.09)	0.33 (0.11)	0.30 (0.09)	0.33 (0.11)	0.33 (0.11)	0.31 (0.10)
Date of COVID-19 diagnosis						
April 1 to June 21, 2022	3762 (17)	9587 (24)	649 (12)	9374 (24)	143 (22)	951 (18)
June 22 to November 30, 2022	13 190 (58)	22 041 (56)	2778 (52)	21 825 (56)	372 (58)	3033 (57)
December 1 to February 20, 2023	5642 (25)	8102 (20)	1884 (36)	8152 (21)	130 (20)	1384 (26)

^a^ Untreated denotes the patients who were eligible for the treatment but did not receive any COVID-19 treatments.

^b^ Other includes American Indian, Alaska Native, Asian, Native Hawaiian, Pacific Islander, and multiracial.

### Nirmatrelvir Use

**Figure 2.**
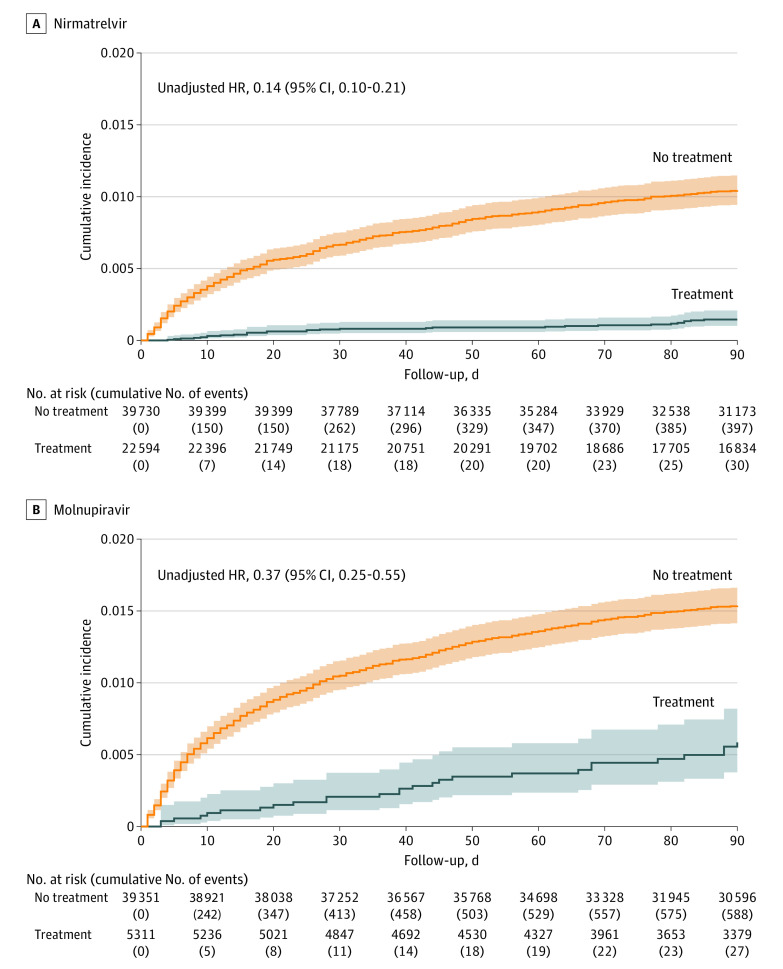
Cumulative Incidence of Death in Patients Infected With Omicron, by Treatment Graphs show incidence of death for patients who received nirmatrelvir (A) and molnupiravir (B) compared with those who were eligible for each treatment but did not receive any treatment. The solid curves show the Kaplan-Meier estimates for the cumulative incidence of death over 90 days after diagnosis of COVID-19. The shaded areas indicate the 95% CIs. The unadjusted hazard ratios (HRs) and 95% CIs are also shown.

Figure 2A shows the cumulative incidence of death for patients treated with nirmatrelvir vs those who were eligible for nirmatrelvir but did not receive any treatment. The cumulative incidence of death at 90 days after COVID-19 diagnosis was 0.15% (95% CI, 0.10%-0.21%) for treated patients and 1.05% (95% CI, 0.95%-1.15%) for untreated patients (eTable 4 in Supplement 1). Among patients aged 65 years or older, the cumulative incidence of death at 90 days was 0.25% (95% CI, 0.17%-0.37%) for the treated and 2.42% (95% CI, 1.15%-2.67%) for the untreated (eFigure 3A and eTable 4 in Supplement 1). Among patients younger than 65 years, the cumulative incidence of death at 90 days was 0.04% (95% CI, 0.02%-0.11%) for the treated and 0.32% (95% CI, 0.25%-0.40%) for the untreated (eFigure 4A and eTable 4 in Supplement 1).

Table 2 shows the association of nirmatrelvir use with the risk of death, with adjustment for potential confounding variables. The adjusted hazard ratio (HR) of death for nirmatrelvir was 0.16 (95% CI, 0.11-0.23). Among patients aged 65 years or older, the adjusted HR was 0.16 (95% CI, 0.11-0.24); among patients younger than 65 years, the adjusted HR was 0.12 (95% CI, 0.04-0.34) (Table 3).

**Table 2.** Adjusted HRs of Death After Omicron Infection for Treatments and Confounding Variables

Variable	HR (95% CI)	
	Nirmatrelvir (n = 62 324)	Molnupiravir (n = 44 662)
Treatment	0.16 (0.11-0.23)	0.23 (0.16-0.34)
Age group, y		
50-64	3.87 (2.32-6.47)	3.71 (2.32-5.94)
65-74	6.31 (3.81-10.44)	6.77 (4.29-10.69)
≥75 y	18.60 (11.49-30.13)	18.35 (11.80-28.54)
Male sex	1.80 (1.48-2.18)	1.56 (1.33-1.83)
Race and ethnicity		
Hispanic	0.62 (0.34-1.15)	0.60 (0.35-1.01)
Non-Hispanic Black	0.60 (0.42-0.85)	0.69 (0.53-0.89)
Other or unknown^a^	1.43 (1.05-1.95)	1.16 (0.85-1.57)
Ohio residency	1.40 (1.05-1.87)	1.45 (1.13-1.86)
SARS-CoV-2 immunity status		
Vaccine boosters	0.46 (0.38-0.56)	0.55 (0.47-0.65)
Previously infected	0.73 (0.51-1.06)	0.76 (0.56-1.02)
Coexisting conditions		
Respiratory	1.19 (0.96-1.47)	1.25 (1.05-1.48)
Immunocompromised	4.60 (3.75-5.66)	4.67 (3.95-5.53)
Cardiovascular system	1.51 (1.16-1.96)	1.55 (1.23-1.96)
Diabetes	1.19 (0.96-1.48)	1.25 (1.04-1.48)
Obesity	1.14 (0.88-1.48)	1.22 (1.00-1.50)
Other nonrespiratory	3.31 (2.47-4.42)	3.07 (2.36-4.00)
Socioeconomic index score	3.57 (1.30-9.76)	2.91 (1.27-6.68)
Date of COVID-19 diagnosis		
June 22 to November 30, 2022	0.80 (0.63-1.01)	0.84 (0.69-1.02)
December 1 to February 20, 2023	0.79 (0.59-1.06)	0.79 (0.62-1.01)

Abbreviation: HR, hazard ratio.

^a^ Other includes American Indian, Alaska Native, Asian, Native Hawaiian, Pacific Islander, and multiracial.

**Table 3.** Adjusted HRs of Death After Omicron Infection for Treatments by Subgroup

Variable	Nirmatrelvir		Molnupiravir	
	Patients, No. treated/total No.	HR (95% CI)	Patients, No. treated/total No.	HR (95% CI)
Age, y				
12-64	11 291/37 087	0.12 (0.04-0.34)	2085/26 579	0.13 (0.04-0.43)
≥65	11 303/25 237	0.16 (0.11-0.24)	3226/18 083	0.24 (0.16-0.37)
Race and ethnicity				
Non-Hispanic White	18 571/46 113	0.15 (0.10-0.23)	4499/31 878	0.23 (0.15-0.34)
All others	4023/16 211	0.18 (0.07-0.50)	812/12 784	0.23 (0.07-0.73)
Vaccine boosters				
Yes	16 966/36 761	0.13 (0.08-0.21)	4162/24 174	0.21 (0.13-0.34)
No	5628/25 563	0.22 (0.12-0.40)	1149/20 488	0.27 (0.13-0.57)
Previous infection				
Yes	1816/6947	0.32 (0.09-1.08)	444/5467	0.47 (0.17-1.35)
No	20 778/55 377	0.15 (0.10-0.22)	4867/39 195	0.21 (0.14-0.32)
Respiratory disorders				
Yes	3994/11 623	0.07 (0.03-0.20)	1242/8938	0.24 (0.13-0.46)
No	18 600/50 701	0.19 (0.13-0.29)	4069/35 724	0.23 (0.14-0.37)
Immunocompromised disorders				
Yes	1339/3627	0.26 (0.15-0.45)	960/3849	0.22 (0.13-0.37)
No	21 255/58 697	0.11 (0.06-0.19)	4351/40 813	0.21 (0.12-0.38)
Cardiovascular system disorders				
Yes	9472/24 187	0.18 (0.12-0.27)	3045/18 708	0.21 (0.14-0.33)
No	13 122/38 137	0.09 (0.03-0.26)	2266/25 954	0.37 (0.15-0.92)
Diabetes				
Yes	3263/8579	0.20 (0.10-0.38)	1215/7080	0.19 (0.10-0.37)
No	19 331/53 745	0.14 (0.09-0.23)	4096/37 582	0.26 (0.16-0.41)
Obesity				
Yes	3255/8911	0.10 (0.03-0.33)	901/6622	0.23 (0.10-0.53)
No	19 339/53 413	0.17 (0.11-0.25)	4410/38 040	0.23 (0.15-0.36)
Other nonrespiratory disorders				
Yes	10 200/28 240	0.16 (0.11-0.25)	3143/21 713	0.21 (0.14-0.32)
No	12 394/34 084	0.13 (0.05-0.36)	2168/22 949	0.39 (0.14-1.10)
Date of COVID-19 diagnosis				
April 1 to June 21, 2022	3762/13 349	0.08 (0.03-0.26)	649/10 023	0.27 (0.11-0.68)
June 22 to November 30, 2022	13 190/35 231	0.16 (0.10-0.26)	2778/24 603	0.22 (0.13-0.38)
December 1 to February 20, 2023	5642/13 744	0.20 (0.10-0.43)	1884/10 036	0.21 (0.10-0.46)

Abbreviation: HR, hazard ratio.

Older age, male sex, and low socioeconomic status were associated with elevated risk of death. Patients who were immunocompromised or who had cardiovascular diseases or other nonrespiratory diseases also had elevated risk of death. Vaccination and previous infection were associated with reduced risk of death. The association of nirmatrelvir with the risk of death was observed across subgroups defined by race and ethnicity, diagnosis date, vaccination status, previous infection status, and coexisting conditions, although the estimate for previously infected patients had great uncertainty owing to a small number of events (Table 3).

### Molnupiravir Use

Figure 2B shows the cumulative incidence of death for patients treated with molnupiravir vs those who were eligible for molnupiravir but did not receive any treatment. At 90 days after COVID-19 diagnosis, the cumulative incidence of death was 0.60% (95% CI, 0.41%-0.88%) for treated patients and 1.57% (95% CI, 1.43%-1.68%) for untreated patients (eTable 4 in Supplement 1). Among patients aged 65 years or older, the cumulative incidence of death at 90 days was 0.88% (95% CI, 0.59%-1.31%) for the treated and 3.46% (95% CI, 3.12%-3.71%) for the untreated (eFigure 3B and eTable 4 in Supplement 1). Among patients younger than 65 years, the cumulative incidence of death at 90 days was 0.17% (95% CI, 0.05%-0.54%) for the treated and 0.44% (95% CI, 0.36%-0.53% for the untreated (eFigure 4B and eTable 4 in Supplement 1).

Table 2 shows the association of molnupiravir use with the risk of death, with adjustment for potential confounding variables. The adjusted HR of death for molnupiravir was 0.23 (95% CI, 0.16-0.34). Among patients aged 65 years or older, the adjusted HR was 0.24 (95% CI, 0.16-0.37); among patients younger than 65 years, the adjusted HR was 0.13 (95% CI, 0.04-0.43) (Table 3). The association of molnupiravir with the risk of death was observed across subgroups defined by race and ethnicity, diagnosis date, vaccination status, previous infection status, and coexisting conditions, although the estimates for previously infected patients and patients without other nonrespiratory conditions had great uncertainties owing to small numbers of events (Table 3).

### Hospitalization

We also fit the Cox regression model to the composite end point of hospitalization and death (ie, time to the occurrence of either event). The adjusted HR was 0.63 (95% CI, 0.59-0.68) for nirmatrelvir and 0.59 (95% CI, 0.53-0.66) for molnupiravir (eTable 5 in Supplement 1). The association was observed across subgroups defined by age, race and ethnicity, diagnosis date, vaccination status, previous infection status, and coexisting conditions, although the estimates for previously infected patients had great uncertainties owing to small numbers of events (eTable 6 in Supplement 1).

### Model Checking

We checked the proportional hazards assumption by estimating the time-varying HR for each variable. There was no evidence for violation of the assumption (eFigures 5 and 6 in Supplement 1).

## Discussion

In this cohort study, both nirmatrelvir and molnupiravir use were found to be associated with reductions in mortality and hospitalization among patients infected with Omicron who were at high risk for progression to severe disease. The associations of both antiviral drugs with both outcomes were observed consistently across subgroups defined by age, race and ethnicity, date of COVID-19 diagnosis, vaccination status, previous infection status, and coexisting conditions. In particular, both drugs were associated with reductions in hospitalization and death caused by the currently circulating BQ.1.1 and XBB.1.5 strains.

Our findings are consistent with those of the phase 3 clinical trials.^3,4^ However, the clinical trials were conducted among unvaccinated populations with limited natural immunity in the Delta era, whereas our study was conducted in a population including vaccinated and previously infected patients during the more recent Omicron period. With a very large sample size, we were able to obtain precise estimates for the association of both drugs with hospitalization and death, not only for the whole population but also for important subgroups.

As mentioned, a recently completed open-label, platform-adaptive randomized clinical trial^7^ did not find evidence that molnupiravir was associated with reduced frequency of COVID-19–associated hospitalizations or death among vaccinated adults in the United Kingdom. However, the patients in that study were at much lower risk of progressing to severe COVID-19 than ours. There were 103 hospitalizations and 3 deaths among 12 529 patients receiving molnupiravir plus usual care vs 96 hospitalizations and 5 deaths among 12 525 patients receiving only usual care.^7^ Thus, the data suggest that molnupiravir might be associated with reduced mortality, although the number of events was too small to have an accurate estimate of the relative risk. In a study^11^ of nirmatrelvir in standard-risk patients, the primary end point of self-reported, sustained alleviation of all symptoms for 4 consecutive days was not met.

In the NIH COVID-19 treatment guidelines,^12^ nirmatrelvir was the preferred treatment for patients at high risk of progressing to severe COVID-19, with molnupiravir as an alternative therapy to be used only when the preferred therapy was not available, feasible to use, or clinically appropriate. The preference for nirmatrelvir over molnupiravir was the result of a greater reduction in the risk of progression to severe COVID-19 observed in the pivotal clinical trial on nirmatrelvir (89%) than that of molnupiravir (48%), but the data on mortality were very limited, with only 1 death observed in the molnupiravir group vs 9 observed in the placebo group through day 29, and with 0 deaths in the nirmatrelvir group vs 7 deaths in the placebo group through day 28.^3,4^ Our study, which had more patients and longer follow-up (Table 1 and Figure 2), demonstrated that both nirmatrelvir and molnupiravir were associated with reductions in mortality. This finding is particularly important because nirmatrelvir has substantial drug-drug interactions with concomitant medications. Although remdesivir is recommended by the NIH, it was not used for nonhospitalized patients at Cleveland Clinic, mainly because it requires 3 infusions.

Observational studies are important tools for evaluating the association of COVID-19 treatments with mortality. Electronic health records from Cleveland Clinic and other health care systems can be leveraged to yield valuable information about the clinical impact of COVID-19 treatments on new variants.

### Limitations

As in any observational study, unmeasured confounders might have biased our HR estimates. We attempted to minimize confounding biases by restricting the study population to patients who were eligible for treatments and by adjusting for demographic and clinical factors that might be associated with treatment selection and death. Compared with other observational studies,^5,6^ a much larger proportion of eligible patients received treatments in our study, such that selection bias should be smaller in our study.

Patients with no or mild symptoms were unlikely to seek therapy, and physicians tended to prioritize treatments for the patients at the highest risk of clinical progression when the treatment supply was limited. These factors would have attenuated the association. On the other hand, treated patients might be more likely to receive medical care or have other characteristics that reduce their risk of death. Such factors would have led to overestimation of the association. Overall, unmeasured confounders could have biased the HR estimates in both directions. However, very strong unmeasured confounding would be required to fully explain away the association observed in this study.

Our study was conducted in a single health care system. Although the states of Ohio and Florida are broadly representative of the US population and the Cleveland Clinic Health System had a very large number of patients treated with nirmatrelvir or molnupiravir, it would be worthwhile to compare our data with those from other health care systems.

## Conclusions

This study found that the use of either nirmatrelvir or molnupiravir was associated with reductions of mortality and hospitalization in patients infected with Omicron, regardless of age, race and ethnicity, virus strain, vaccination status, previous infection status, or coexisting conditions. Both drugs can, therefore, be used to treat nonhospitalized patients who are at high risk of progressing to severe COVID-19.

## Notes

Supplement 1. **eFigure 1.** Outpatient Covid-19 Therapeutic Decision Tree at Cleveland Clinic **eFigure 2.** Proportions of Patients Receiving Treatments Over Time **eFigure 3.** Cumulative Incidence of Death After Omicron Infection by Treatments for Patients 65 Years of Age or Older **eFigure 4.** Cumulative Incidence of Death After Omicron Infection by Treatments for Patients Less Than 65 Years of Age **eFigure 5.** Estimates of Time-Varying Hazard Ratios for Time to Death in the Cox Regression Analysis of Nirmatrelvir Treatment **eFigure 6.** Estimates of Time-Varying Hazard Ratios for Time to Death in the Cox Regression Analysis of Molnupiravir Treatment **eTable 1.** *ICD-10* Codes for the Underlying Conditions of Covid-19 Patients at High Risk of Progressing to Severe Disease **eTable 2.** Categories of Comorbidities for Covid-19 Patients **eTable 3.** Differences of Demographic and Clinical Characteristics Between Treated and Untreated Patients **eTable 4.** Kaplan-Meier Estimates and 95% Confidence Intervals for the Cumulative Incidence of Death After Covid-19 Diagnosis **eTable 5.** Adjusted Hazard Ratios of Hospitalization or Death After Omicron Infection for Treatments and Confounding Variables **eTable 6.** Adjusted Hazard Ratios of Hospitalization or Death After Omicron Infection for Treatments by Subgroup

Supplement 2.
Data Sharing Statement
