# Supplementary material for: Nirmatrelvir or Molnupiravir Use and Severe Outcomes From Omicron Infections
Source: JAMA Netw Open. 2023 Sep 21;6(9):e2335077. doi: 10.1001/jamanetworkopen.2023.35077 (PMC10514733; doi:10.1001/jamanetworkopen.2023.35077)
Supplement: Supplement 1. — eFigure 1. Outpatient Covid-19 Therapeutic Decision Tree at Cleveland Clinic eFigure 2. Proportions of Patients Receiving Treatments Over Time eFigure 3. Cumulative Incidence of Death After Omicron Infection by Treatments for Patients 65 Years of Age or Older eFigure 4. Cumulative Incidence of Death After Omicron Infection by Treatments for Patients Less Than 65 Years of Age eFigure 5. Estimates of Time-Varying Hazard Ratios for Time to Death in the Cox Regression Analysis of Nirmatrelvir Treatment eFigure 6. Estimates of Time-Varying Hazard Ratios for Time to Death in the Cox Regression Analysis of Molnupiravir Treatment eTable 1. ICD-10 Codes for the Underlying Conditions of Covid-19 Patients at High Risk of Progressing to Severe Disease eTable 2. Categories of Comorbidities for Covid-19 Patients eTable 3. Differences of Demographic and Clinical Characteristics Between Treated and Untreated Patients eTable 4. Kaplan-Meier Estimates and 95% Confidence Intervals for the Cumulative Incidence of Death After Covid-19 Diagnosis eTable 5. Adjusted Hazard Ratios of Hospitalization or Death After Omicron Infection for Treatments and Confounding Variables eTable 6. Adjusted Hazard Ratios of Hospitalization or Death After Omicron Infection for Treatments by Subgroup [file jamanetwopen-e2335077-s001.pdf]

Supplemental Online Content

Lin DY, Abi Fadel F, Huang S, et al. Nirmatrelvir or molnupiravir use and severe outcomes from Omicron infections. *JAMA Netw Open*. 2023;6(9):e2335077. doi:10.1001/jamanetworkopen.2023.35077

eFigure 1. Outpatient Covid-19 Therapeutic Decision Tree at Cleveland Clinic
eFigure 2. Proportions of Patients Receiving Treatments Over Time
eFigure 3. Cumulative Incidence of Death After Omicron Infection by Treatments for Patients 65 Years of Age or Older
eFigure 4. Cumulative Incidence of Death After Omicron Infection by Treatments for Patients Less Than 65 Years of Age
eFigure 5. Estimates of Time-Varying Hazard Ratios for Time to Death in the Cox Regression Analysis of Nirmatrelvir Treatment
eFigure 6. Estimates of Time-Varying Hazard Ratios for Time to Death in the Cox Regression Analysis of Molnupiravir Treatment
eTable 1. *ICD-10* Codes for the Underlying Conditions of Covid-19 Patients at High Risk of Progressing to Severe Disease
eTable 2. Categories of Comorbidities for Covid-19 Patients
eTable 3. Differences of Demographic and Clinical Characteristics Between Treated and Untreated Patients
eTable 4. Kaplan-Meier Estimates and 95% Confidence Intervals for the Cumulative Incidence of Death After Covid-19 Diagnosis
eTable 5. Adjusted Hazard Ratios of Hospitalization or Death After Omicron Infection for Treatments and Confounding Variables
eTable 6. Adjusted Hazard Ratios of Hospitalization or Death After Omicron Infection for Treatments by Subgroup

This supplemental material has been provided by the authors to give readers additional information about their work.

© 2023 Lin DY et al. *JAMA Network Open.*

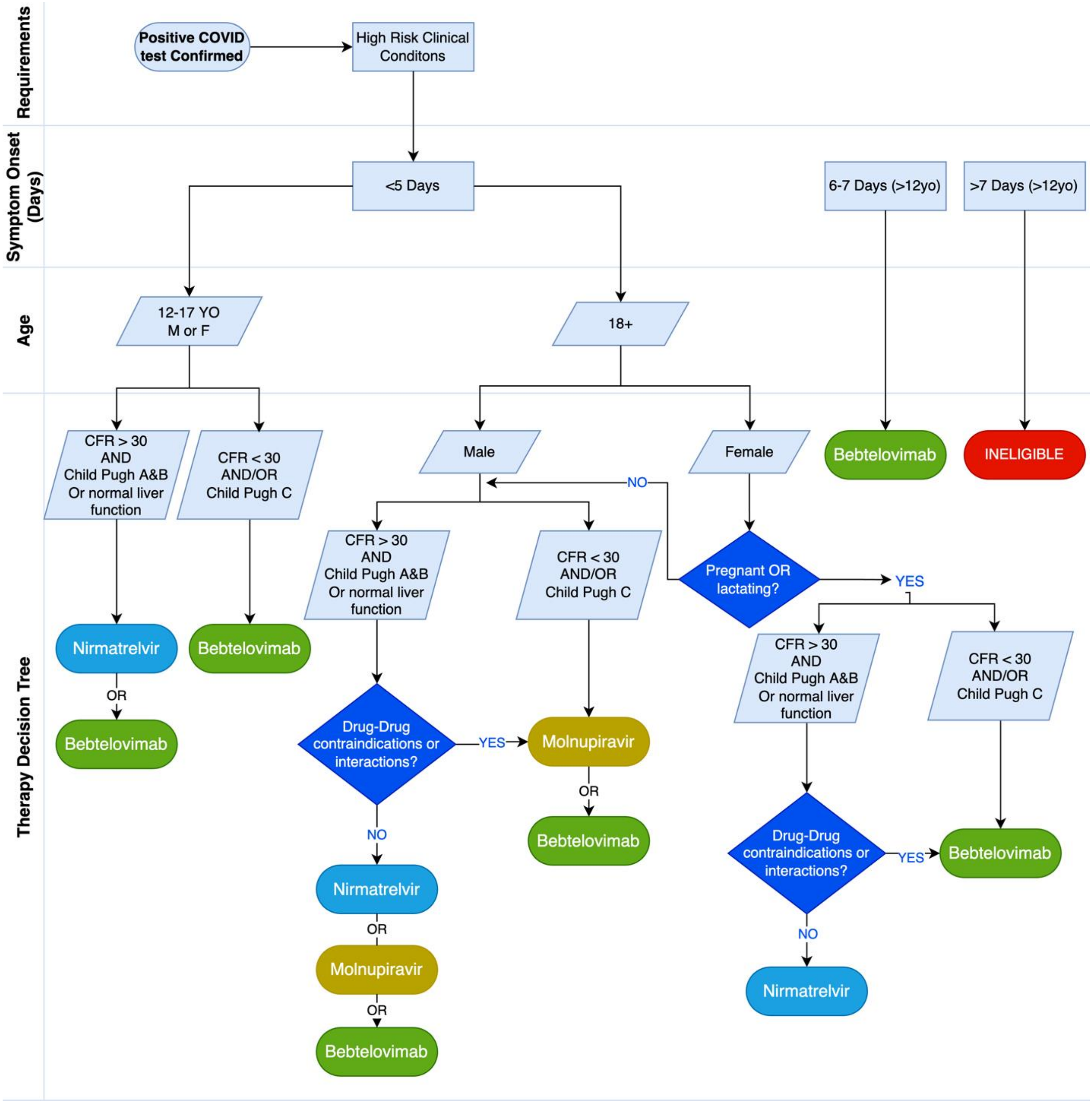

eFigure 1. Outpatient Covid-19 Therapeutic Decision Tree at Cleveland Clinic.

© 2023 Lin DY et al. *JAMA Network Open.*

A Nirmatrelvir

B Molnupiravir

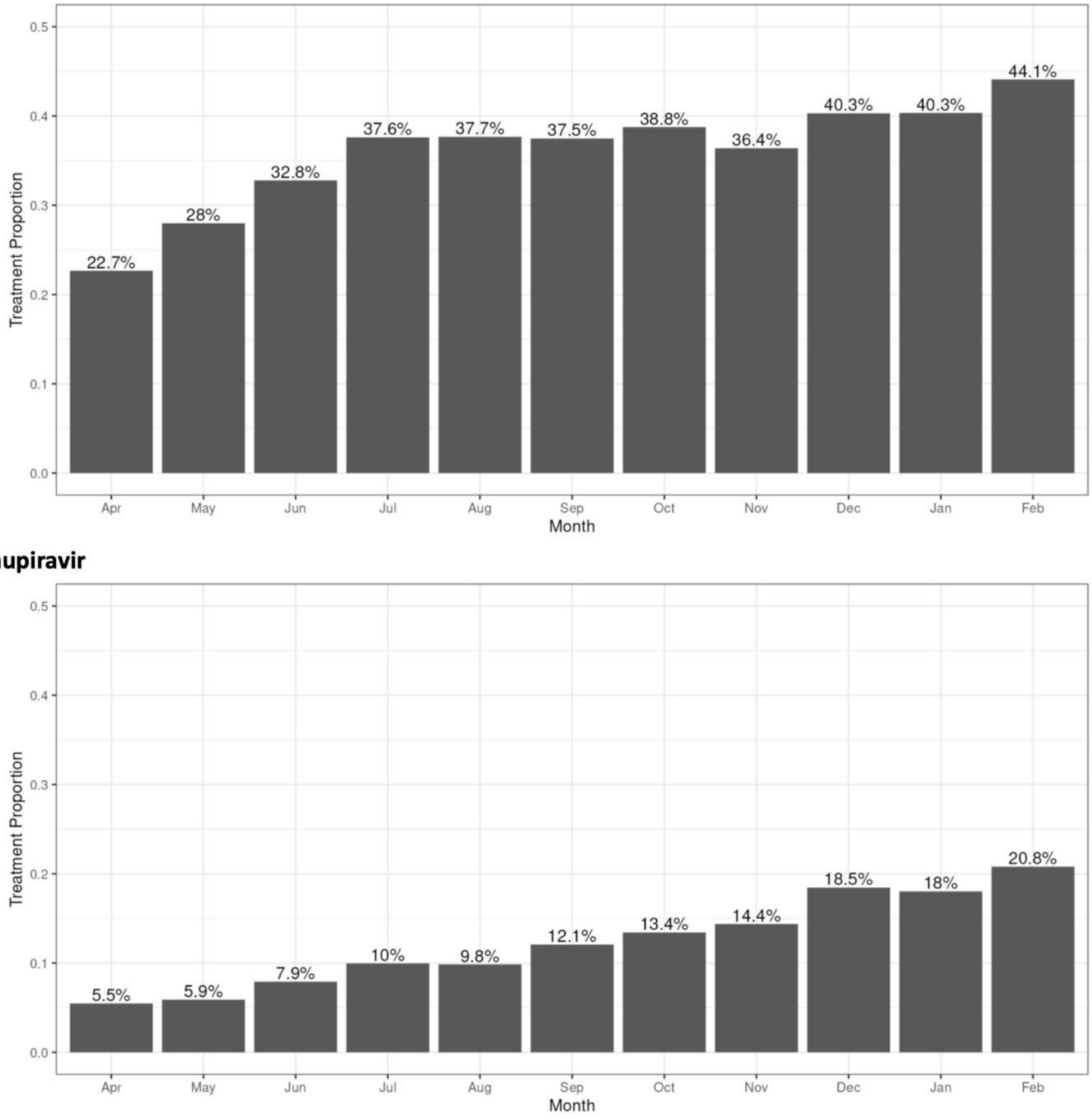

eFigure 2. Proportions of Patients Receiving Treatments Over Time

© 2023 Lin DY et al. *JAMA Network Open.*

A Nirmatrelvir

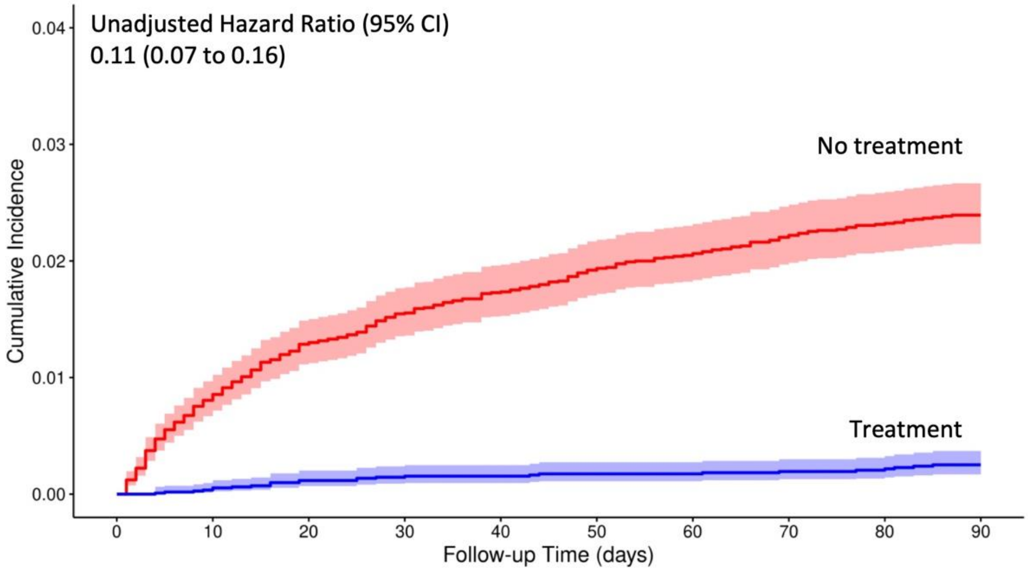

No. at Risk
(Cumulative No. of Events)

No Treatment	13,934 (0)	13,733 (119)	13,386 (180)	13,114 (214)	12,858 (238)	12,566 (264)	12,152 (280)	11,693 (299)	11,214 (311)	10,760 (319)
Treatment	11,303 (0)	11,186 (6)	10,866 (13)	10,614 (17)	10,407 (17)	10,164 (19)	9,865 (19)	9,344 (21)	8,856 (23)	8,422 (26)

B Molnupiravir

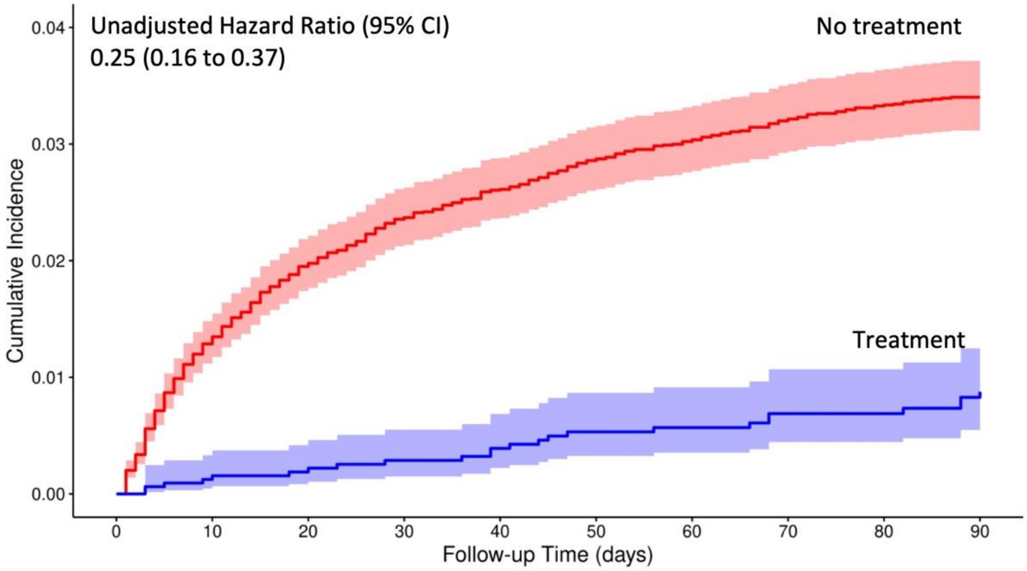

No. at Risk
(Cumulative No. of Events)

No Treatment	14,857 (0)	14,566 (200)	14,165 (292)	13,851 (348)	13,565 (382)	13,245 (418)	12,796 (440)	12,299 (463)	11,785 (478)	11,300 (486)
Treatment	3,226 (0)	3,178 (5)	3,055 (7)	2,961 (9)	2,869 (12)	2,776 (16)	2,638 (17)	2,415 (20)	2,231 (20)	2,063 (24)

eFigure 3. Cumulative Incidence of Death After Omicron Infection by Treatments for Patients 65 Years of Age or Older.

© 2023 Lin DY et al. *JAMA Network Open.*

A Nirmatrelvir

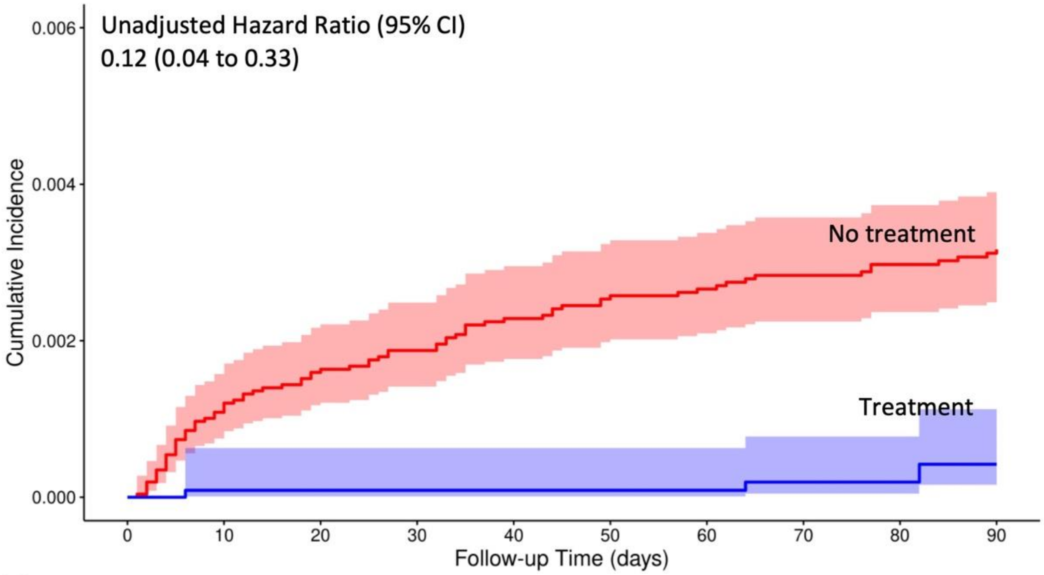

No. at Risk
(Cumulative No. of Events)

	0	10	20	30	40	50	60	70	80	90
No Treatment	25,796 (0)	25,666 (31)	25,165 (42)	24,675 (48)	24,256 (58)	23,769 (65)	23,132 (67)	22,236 (71)	21,324 (74)	20,413 (78)
Treatment	11,291 (0)	11,210 (1)	10,883 (1)	10,561 (1)	10,344 (1)	10,127 (1)	9,837 (1)	9,342 (2)	8,849 (2)	8,412 (4)

B Molnupiravir

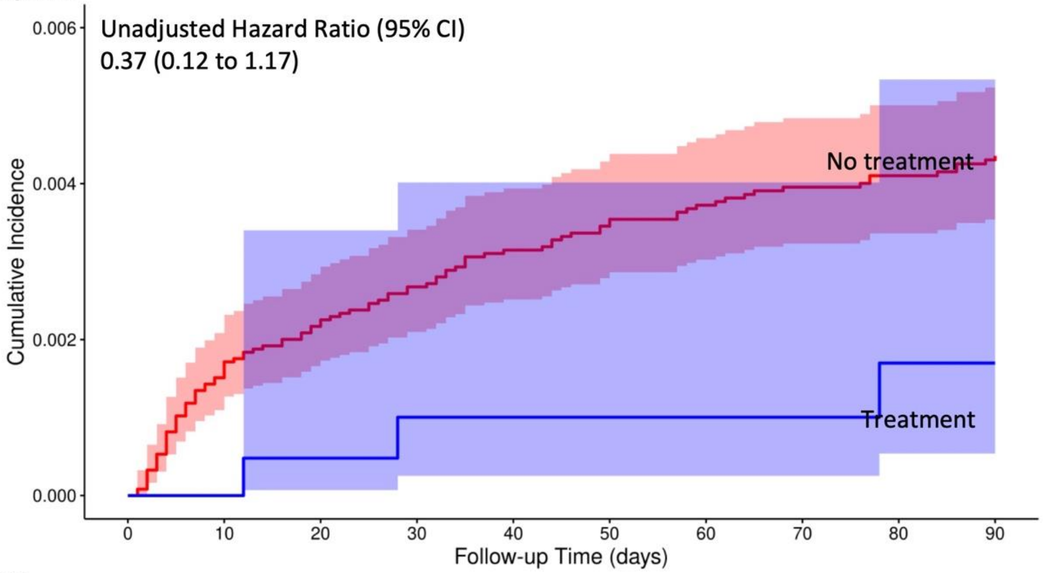

No. at Risk
(Cumulative No. of Events)

	0	10	20	30	40	50	60	70	80	90
No Treatment	24,494 (0)	24,457 (42)	23,873 (55)	23,401 (65)	23,002 (76)	22,523 (85)	21,902 (89)	21,029 (94)	20,160 (97)	19,296 (102)
Treatment	2,085 (0)	2,085 (0)	1,966 (1)	1,886 (2)	1,823 (2)	1,754 (2)	1,689 (2)	1,546 (2)	1,422 (3)	1,316 (3)

eFigure 4. Cumulative Incidence of Death After Omicron Infection by Treatments for Patients Less Than 65 Years of Age.

© 2023 Lin DY et al. *JAMA Network Open.*

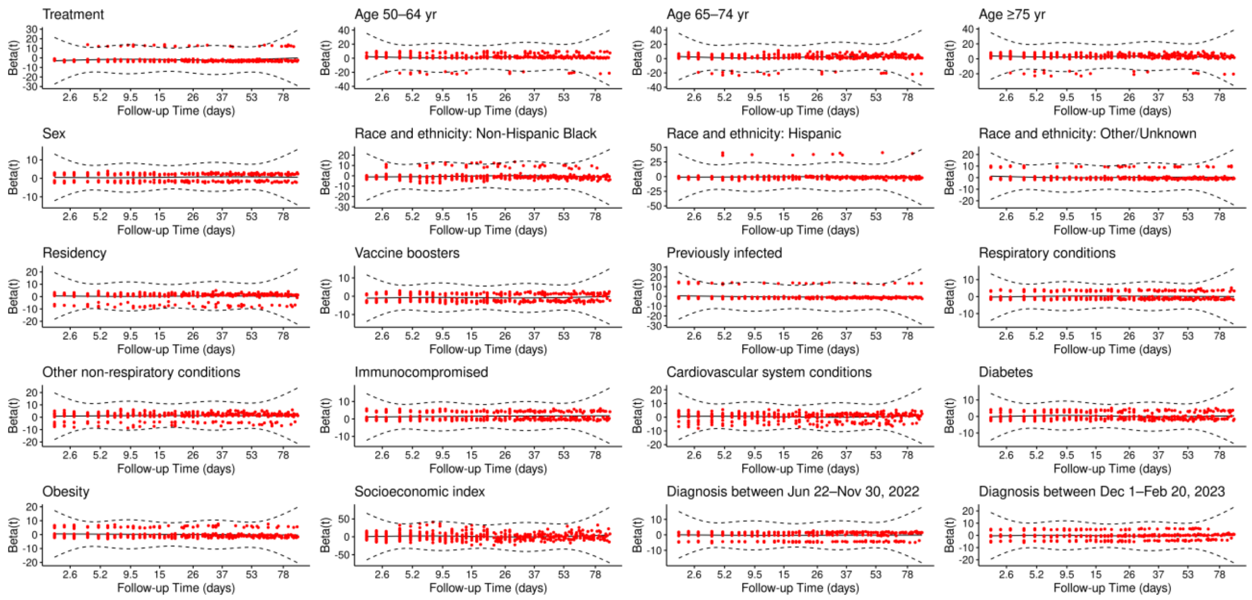

eFigure 5. Estimates of Time-Varying Hazard Ratios for Time to Death in the Cox Regression Analysis of Nirmatrelvir Treatment.

© 2023 Lin DY et al. *JAMA Network Open.*

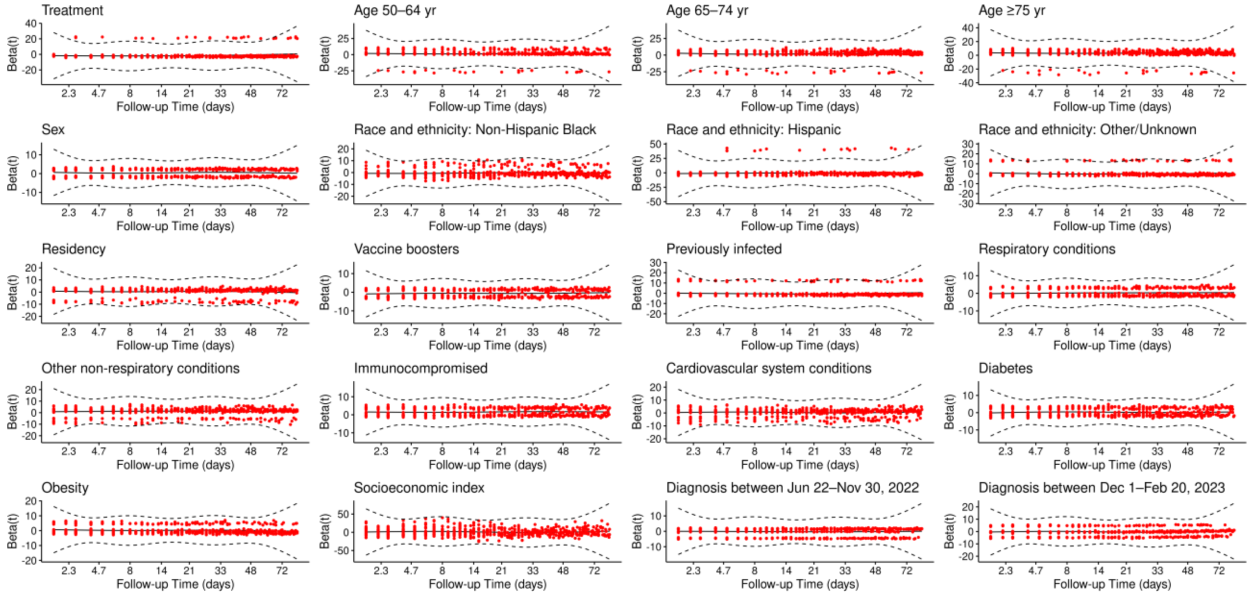

eFigure 6. Estimates of Time-Varying Hazard Ratios for Time to Death in the Cox Regression Analysis of Molnupiravir Treatment.

© 2023 Lin DY et al. *JAMA Network Open.*

eTable 1. ***ICD-10*** **Codes for the Underlying Conditions of Covid-19 Patients at High Risk of Progressing to Severe Disease.**

Conditions	ICD-10 diagnosis code
Overweight (BMI>30) and obesity	E66
Diabetes mellitus	E08-E13
Pregnancy, childbirth and the puerperium	O00-O9A
Glomerular diseases	N00-N08
Renal tubulo-interstitial diseases	N10-N16
Acute kidney failure and chronic kidney disease	N17-N19
Diseases of the circulatory system	I00-I99
Diseases of the respiratory system	J40-J99
Sickle-cell disorders	D57
Diseases of the nervous system	G00-G99
Congenital malformations of the nervous system	Q00-Q07
Artificial opening status	Z93
Neutropenia	D70
Functional disorders of polymorphonuclear neutrophils	D71
Certain disorders involving the immune mechanism	D80-D89
Long term (current) use of systemic steroids	Z79.52
Human immunodeficiency virus [HIV] disease	B20
Autoinflammatory syndromes	M04-M04
Inflammatory polyarthropathies	M05-M14
Systemic connective tissue disorders	M30-M36
Myositis	M60
Ankylosing spondylitis	M45
Other inflammatory spondylopathies	M46
Lupus erythematosus	L93
Other localized connective tissue disorders	L94
Vasculitis limited to skin, not elsewhere classified	L95
Neoplasms	C00-D49
Encounter for antineoplastic radiation therapy	Z51.0
Encounter for antineoplastic chemotherapy and immunotherapy	Z51.1
Encounter for antineoplastic chemotherapy	Z51.11
Encounter for antineoplastic immunotherapy	Z51.12

© 2023 Lin DY et al. *JAMA Network Open.*

eTable 2. Categories of Comorbidities for Covid-19 Patients.[a]

**Respiratory conditions**	Chronic pulmonary disease<br>Pulmonary circulation disease
**Immunocompromised conditions**[b]	Human immunodeficiency virus<br>Pneumocystosis<br>Malignant neoplasm associated with transplanted organ<br>Other malignant immunoproliferative diseases
**Cardiovascular system conditions**	Congestive heart failure<br>Valvular disease<br>Peripheral vascular disease<br>Hypertensive disease<br>Ischemic heart disease<br>Cerebrovascular disease
**Diabetes conditions**	Diabetes without chronic complications<br>Diabetes with chronic complications
**Obesity conditions**	Obesity
**Other non-respiratory conditions**	Paralysis<br>Other neurological disorders<br>Hypothyroidism<br>Renal failure<br>Liver disease<br>Peptic ulcer Disease x bleeding<br>Lymphoma<br>Metastatic cancer<br>Solid tumor w/out metastasis<br>Rheumatoid arthritis/collagen vas<br>Coagulopthy<br>Fluid and electrolyte disorders<br>Chronic blood loss anemia<br>Deficiency Anemias<br>Alcohol abuse<br>Drug abuse<br>Psychoses<br>Depression

a. The Elixhauser Comorbidity Software, Version 3.7 was used to extract the comorbidity variables. The comorbidity was based on diagnosis within 30 days of the COVID diagnosis date and followed the definition from "The AHRQ Elixhauser Comorbidity index. Med Care. 2017 Jul; 55(7):698-705". The definitions and ICD-10 codes of the comorbidity variables can be found at
https://www.hcup-us.ahrq.gov/toolssoftware/comorbidity/comorbidity.jsp

b. The full list of immunocompromised states is available at
https://www.qualityindicators.ahrq.gov/Downloads/Modules/PSI/V60-ICD10/TechSpecs/PSI_Appendix_I.pdf

© 2023 Lin DY et al. *JAMA Network Open.*

eTable 3. Differences of Demographic and Clinical Characteristics Between Treated and Untreated Patients

Variable	Mean Difference (95% CI)	
	Nirmatrelvir	Molnupiravir
Age	7.86 (7.59 to 8.14)	9.99 (9.56 to 10.43)
Female sex (%)	-3.09 (-3.88 to -2.29)	-9.45 (-10.89 to -8.01)
Race and ethnicity (%)		
Hispanic	-2.32 (-2.69 to -1.96)	-3.03 (-3.58 to -2.48)
non-Hispanic Black	-8.95 (-9.44 to -8.46)	-10.13 (-10.88 to -9.38)
Non-Hispanic White	12.87 (12.19 to 13.55)	15.13 (14.05 to 16.21)
Ohio residency (%)	0.39 (-0.17 to 0.95)	1.44 (0.47 to 2.41)
SARS-CoV-2 immunity status (%)		
Vaccine boosters	25.27 (24.52 to 26.02)	27.51 (26.29 to 28.73)
Previously infected	-4.88 (-5.36 to -4.39)	-4.40 (-5.23 to -3.58)
Coexisting conditions (%)		
Respiratory	-1.52 (-2.16 to -0.89)	3.83 (2.61 to 5.04)
Immunocompromised	0.17 (-0.22 to 0.55)	10.73 (9.66 to 11.81)
Cardiovascular system	4.89 (4.08 to 5.69)	17.53 (16.10 to 18.96)
Diabetes	1.06 (0.49 to 1.63)	7.97 (6.78 to 9.17)
Obesity	0.17 (-0.41 to 0.75)	2.43 (1.35 to 3.50)
Other non-respiratory	-0.26 (-1.08 to 0.55)	11.99 (10.57 to 13.41)
Socioeconomic index	-0.03 (-0.03 to -0.03)	-0.03 (-0.03 to -0.03)
Covid-19 diagnosis (%)		
Apr 1– Jun 21, 2022	-7.48 (-8.13 to -6.83)	-11.60 (-12.59 to -10.61)
Jun 22 – Nov 30, 2022	2.90 (2.09 to 3.71)	-3.16 (-4.60 to -1.71)

© 2023 Lin DY et al. *JAMA Network Open.*

eTable 4. Kaplan-Meier Estimates and 95% Confidence Intervals for the Cumulative Incidence of Death After Covid-19 Diagnosis

	30 Days		60 Days		90 Days	
	Treatment	No Treatment	Treatment	No treatment	Treatment	No treatment
**Nirmatrelvir**						
All Patients	0.08%<br>(0.05 to 0.13)	0.67%<br>(0.59 to 0.75)	0.09%<br>(0.06 to 0.14)	0.90%<br>(0.81 to 0.99)	0.15%<br>(0.10 to 0.21)	1.05%<br>(0.95 to 1.15)
≥65 yrs	0.15%<br>(0.10 to 0.25)	1.57%<br>(1.36 to 1.77)	0.17%<br>(0.11 to 0.27)	2.08%<br>(1.84 to 2.32)	0.25%<br>(0.17 to 0.37)	2.42%<br>(1.15 to 2.67)
<65 yrs	0.01%<br>(0.001 to 0.06)	0.19%<br>(0.14 to 0.25)	0.01%<br>(0.008 to 0.06)	0.27%<br>(0.21 to 0.34)	0.04%<br>(0.02 to 0.11)	0.32%<br>(0.25 to 0.40)
**Molnupiravir**						
All Patients	0.21%<br>(0.12 to 0.39)	1.07%<br>(0.96 to 1.17)	0.39%<br>(0.25 to 0.61)	1.39%<br>(1.26 to 1.50)	0.60%<br>(0.41 to 0.88)	1.57%<br>(1.43 to 1.68)
≥65 yrs	0.29%<br>(0.15 to 0.55)	2.40%<br>(2.14 to 2.63)	0.57%<br>(0.35 to 0.91)	3.08%<br>(2.77 to 3.33)	0.88%<br>(0.59 to 1.31)	3.46%<br>(3.12 to 3.71)
<65 yrs	0.10%<br>(0.03 to 0.40)	0.27%<br>(0.21 to 0.34)	0.10%<br>(0.03 to 0.40)	0.37%<br>(0.30 to 0.46)	0.17%<br>(0.05 to 0.54)	0.44%<br>(0.36 to 0.53)

© 2023 Lin DY et al. *JAMA Network Open.*

eTable 5. Adjusted Hazard Ratios of Hospitalization or Death After Omicron Infection for Treatments and Confounding Variables

**Variable**	**Hazard Ratio (95% CI)**	
	Nirmatrelvir (N=62,324)	Molnupiravir (N=44,662)
Treatment	0.63 (0.59 to 0.68)	0.59 (0.53 to 0.66)
Age group		
50–64 yr	1.10 (1.01 to 1.20)	1.20 (1.09 to 1.32)
65–74 yr	1.20 (1.10 to 1.31)	1.39 (1.26 to 1.53)
≥75 yr	1.54 (1.40 to 1.68)	1.78 (1.62 to 1.96)
Male sex	0.99 (0.93 to 1.05)	1.06 (1.00 to 1.13)
Race and ethnicity		
Hispanic	0.86 (0.74 to 1.00)	0.88 (0.76 to 1.03)
Black, non-Hispanic	0.87 (0.79 to 0.95)	0.89 (0.81 to 0.98)
Other/Unknown	1.04 (0.93 to 1.17)	1.01 (0.89 to 1.14)
Ohio residency	1.62 (1.46 to 1.81)	1.41 (1.28 to 1.57)
SARS-CoV-2 immunity status		
Vaccine boosters	0.82 (0.77 to 0.87)	0.79 (0.74 to 0.84)
Previously infected	0.95 (0.87 to 1.04)	0.96 (0.87 to 1.05)
Coexisting conditions		
Respiratory	1.38 (1.29 to 1.47)	1.32 (1.23 to 1.41)
Immunocompromised	2.24 (2.07 to 2.43)	2.39 (2.22 to 2.57)
Cardiovascular system	1.57 (1.47 to 1.69)	1.65 (1.53 to 1.78)
Diabetes	1.12 (1.04 to 1.20)	1.10 (1.03 to 1.19)
Obesity	1.44 (1.34 to 1.54)	1.44 (1.34 to 1.55)
Other non-respiratory	2.42 (2.26 to 2.59)	2.52 (2.33 to 2.73)
Socioeconomic index	0.54 (0.40 to 0.74)	0.64 (0.47 to 0.88)
Covid-19 diagnosis		
Jun 22 – Nov 30, 2022	1.06 (0.98 to 1.14)	1.09 (1.00 to 1.18)
Dec 1– Feb 20, 2023	1.21 (1.11 to 1.32)	1.24 (1.13 to 1.36)

© 2023 Lin DY et al. *JAMA Network Open.*

eTable 6. Adjusted Hazard Ratios of Hospitalization or Death After Omicron Infection for Treatments by Subgroup.

	Nirmatrelvir		Molnupiravir	
	No. treated/N	Hazard Ratio (95% CI)	No. treated/N	Hazard Ratio (95% CI)
Age				
12–64 yr	11,291/37,087	0.67 (0.61 to 0.75)	2,085/26,579	0.58 (0.48 to 0.70)
≥65 yr	11,303/25,237	0.60 (0.55 to 0.66)	3,226/18,083	0.60 (0.53 to 0.68)
Race and ethnicity				
Non-Hispanic White	18,571/46,113	0.63 (0.59 to 0.68)	4,499/31,878	0.60 (0.54 to 0.67)
Others	4,023/16,211	0.61 (0.52 to 0.73)	812/12,784	0.54 (0.41 to 0.71)
Vaccine boosters				
Yes	16,966/36,761	0.63 (0.58 to 0.69)	4,162/24,174	0.59 (0.52 to 0.66)
No	5,628/25,563	0.58 (0.51 to 0.66)	1,149/20,488	0.56 (0.45 to 0.71)
Previous infection				
Yes	1,816/6,947	0.70 (0.56 to 0.86)	444/5,467	0.75 (0.55 to 1.02)
No	20,778/55,377	0.63 (0.58 to 0.67)	4,867/39,195	0.57 (0.51 to 0.64)
Respiratory disorders				
Yes	3,994/11,623	0.71 (0.62 to 0.80)	1,242/8,938	0.63 (0.53 to 0.76)
No	18,600/50,701	0.60 (0.56 to 0.66)	4,069/35,724	0.57 (0.50 to 0.65)
Immunocompromised disorders				
Yes	1,339/3,627	0.54 (0.45 to 0.66)	960/3,849	0.48 (0.39 to 0.58)
No	21,255/58,697	0.65 (0.60 to 0.70)	4,351/40,813	0.66 (0.58 to 0.75)
Cardiovascular system disorders				
Yes	9,472/24,187	0.60 (0.55 to 0.66)	3,045/18,708	0.56 (0.50 to 0.64)
No	13,122/38,137	0.69 (0.62 to 0.78)	2,266/25,954	0.71 (0.57 to 0.87)
Diabetes				
Yes	3,263/8,579	0.65 (0.57 to 0.76)	1,215/7,080	0.52 (0.43 to 0.63)
No	19,331/53,745	0.63 (0.58 to 0.68)	4,096/37,582	0.63 (0.55 to 0.71)
Obesity				
Yes	3,255/8,911	0.56 (0.48 to 0.64)	901/6,622	0.54 (0.43 to 0.66)
No	19,339/53,413	0.66 (0.60 to 0.71)	4,410/38,040	0.61 (0.54 to 0.69)
Other non-respiratory disorders				
Yes	10,200/28,240	0.62 (0.57 to 0.67)	3,143/21,713	0.56 (0.50 to 0.63)
No	12,394/34,084	0.68 (0.59 to 0.77)	2,168/22,949	0.76 (0.60 to 0.96)
Covid-19 diagnosis				
Apr 1– Jun 21, 2022	3,762/13,349	0.66 (0.56 to 0.78)	649/10,023	0.62 (0.46 to 0.83)
Jun 22 – Nov 30, 2022	13,190/35,231	0.65 (0.60 to 0.71)	2,778/24,603	0.59 (0.51 to 0.68)
Dec 1– Feb 20, 2023	5,642/13,744	0.57 (0.49 to 0.65)	1,884/10,036	0.57 (0.47 to 0.68)

© 2023 Lin DY et al. *JAMA Network Open.*
