# Supplementary material for: Nirmatrelvir or Molnupiravir Use and Severe Outcomes From Omicron Infections
Source: JAMA Netw Open. 2023 Sep 21;6(9):e2335077. doi: 10.1001/jamanetworkopen.2023.35077 (PMC10514733; doi:10.1001/jamanetworkopen.2023.35077)
Supplement: Supplement 2. — Data Sharing Statement [file jamanetwopen-e2335077-s002.pdf]

Data Sharing Statement

Lin. Nirmatrelvir or Molnupiravir Use and Severe Outcomes From Omicron Infections. *JAMA Netw Open*. Published September 21, 2023. doi:10.1001/jamanetworkopen.2023.35077

Data
Data available: No

Additional Information
Explanation for why data not available: Patient data cannot be shared with third parties per institutional policies.
